# Supplementary material for: Complexome profiling of the Chlamydomonas psb28 mutant reveals TEF5 as an early PSII assembly factor
Source: Plant Cell. 2025 Mar 17;37(6):koaf055. doi: 10.1093/plcell/koaf055 (PMC12164590; doi:10.1093/plcell/koaf055)
Supplement: koaf055_Supplementary_Data [file koaf055_supplementary_data.zip › psb28_interactive.zip!/plots/intensity/qualityPlots/peptides/PDY1_Cre01.g036850.t1.2.html]

### Cre01.g036850.t1.2

Trivial name: PDY1  
SeqCoverage [median]: 20.30  
Mass: 65.92 kDa  
  
 

| Sequence | MissedCleavage | Mass [Da] | wtIntensity | muIntensity | Score |
| --- | --- | --- | --- | --- | --- |
| AELMVGTHNR | 0 | 1126.56 | 4120.88 | 4604.82 | 67.11 |
| ASAGAAAGGAAQVSAR | 0 | 1314.66 | 2217.81 | 2021.64 | 157.50 |
| DPLGGESLIESGVAAR | 0 | 1569.80 | 4716.06 | 847.66 | 204.48 |
| ESQFTFDDHQAIFEGR | 0 | 1925.85 | 0.00 | 975.20 | 59.86 |
| INEAQYTR | 0 | 993.49 | 550.69 | 0.00 | 83.27 |
| LRDPAHSPIAR | 1 | 1231.68 | 1023.29 | 0.00 | 56.40 |
| LVAQAVK | 0 | 727.46 | 631.60 | 0.00 | 89.90 |
| QELESQTDAR | 0 | 1175.54 | 0.00 | 294.32 | 142.98 |
| QGFLEAFER | 0 | 1095.53 | 1243.54 | 0.00 | 56.73 |
| RLPDMAASLR | 1 | 1128.61 | 289.84 | 0.00 | 45.88 |
| SSVEGVIER | 0 | 974.50 | 2475.62 | 0.00 | 75.38 |
|  |  |  | 17269.3 | 8743.6 |  |
